# Recent Advances in Organic Piezoelectric Biomaterials for Energy and Biomedical Applications

**DOI:** 10.3390/nano10010123

**Published:** 2020-01-09

**Authors:** Dong-Myeong Shin, Suck Won Hong, Yoon-Hwae Hwang

**Affiliations:** 1 Department of Mechanical Engineering, The University of Hong Kong, Hong Kong 999077, China; 2 Department of Cogno-Mechatronics Engineering, Department of Optics and Mechatronics Engineering, Pusan National University (PNU), Busan 46241, Korea; swhong@pusan.ac.kr; 3 Department of Nanoenergy Engineering & BK21 PLUS Nanoconvergence Technology Division, Pusan National University (PNU), Busan 46241, Korea; yhwang@pusan.ac.kr

**Keywords:** piezoelectric materials, organic materials, biomaterials, energy applications, biomedical applications

## Abstract

The past decade has witnessed significant advances in medically implantable and wearable devices technologies as a promising personal healthcare platform. Organic piezoelectric biomaterials have attracted widespread attention as the functional materials in the biomedical devices due to their advantages of excellent biocompatibility and environmental friendliness. Biomedical devices featuring the biocompatible piezoelectric materials involve energy harvesting devices, sensors, and scaffolds for cell and tissue engineering. This paper offers a comprehensive review of the principles, properties, and applications of organic piezoelectric biomaterials. How to tackle issues relating to the better integration of the organic piezoelectric biomaterials into the biomedical devices is discussed. Further developments in biocompatible piezoelectric materials can spark a new age in the field of biomedical technologies.

## 1. Introduction

Piezoelectric materials are a class of solid materials that can accumulate an electric charge in response to applied mechanical agitation, facilitating the conversion from mechanical energy to electrical energy and vice versa. Piezoelectricity has been found in both organic and inorganic materials, where the physical principles of piezoelectricity are varied upon material classification. In inorganic piezoelectric materials, the piezoelectric effect arises from the rearrangement of ions in the dielectric materials that possess a lack of inversion symmetry in crystalline structure [1]. In contrast, the reorientation of molecular dipole mainly induces polarization in organic piezoelectric materials under applied mechanical stress [2,3]. These materials have taken over the entire market of electromechanical devices, such as sensors [4,5,6], actuators [7], energy harvesting [8,9,10] and storage [11,12]. Recently, medically implantable and mountable devices have attracted considerable attention [13,14], and are the newly emerging applications for piezoelectric materials.

Organic piezoelectric biomaterials offer several benefits over inorganic piezoelectric materials, which include a high biocompatibility, excellent flexibility, environmental friendliness, and a high level of processability. Ever since the discovery of polarization in asymmetric biological tissue in 1941 [15], many researchers have looked not only to unveil the primary principle underlying the piezoelectricity of those materials, but also to enhance its physical and chemical properties by designing a molecular structure, nanostructuring, and adding dopants [2,16]. Although organic piezoelectric biomaterials exhibit weak piezoelectricity compared to inorganic counterparts, recent research suggests that biocompatible piezoelectric materials, which are interfaced with the biological system of human beings, can serve as the functional materials in the field of medically implantable and mountable applications when they are well-processed. Organic piezoelectric materials are applicable in broad range of devices, including nano- to millimeter-scaled devices, so there might be some challenges in the device fabrication due to local damage and nonlocal elasticity [17,18]. However, our manuscript will only detail views on organic piezoelectric materials.

The rapid development in organic piezoelectric biomaterials calls for a comprehensive review that can provide a useful reference for researchers in relevant fields. Herein, we provide a thorough review of organic piezoelectric biomaterials that are used in energy and biomedical applications. We review the working principle and properties of the different types of organic piezoelectric biomaterials. Efforts to improve the piezoelectric performance of each materials are discussed. The applications of these materials are introduced in terms of energy harvesting, sensor, and cell and tissue regeneration. Meanwhile, the challenges that need to be addressed for practical application are also presented.

## 2. Mechanism of Piezoelectricity in Biomaterials

Piezoelectricity in organic biomaterials mainly originated from the reorientation of the molecular dipole [2,3] while the breaking of structural symmetry in crystal lattices results in piezoelectricity in traditional inorganic materials [1]. As a piezoelectric biomaterial is deformed under stress, the molecular chains with a permanent dipole in the material are aligned along one direction, yielding or changing in net polarization so that it is able to represent the piezoelectric behavior regardless of the absence of non-centrosymmetry. Therefore, the piezoelectric biomaterials are required to possess the presence of permanent molecular dipoles, the ability to orient the molecular dipoles, and the ability to maintain the dipole alignment [19]. The following sections provide detail on piezoelectric mechanisms and the properties of these materials, including the proteins, peptides, and biopolymers, and the piezoelectric constants summarized in Table 1.

### 2.1. Piezoelectric Proteins

**Figure 1:**
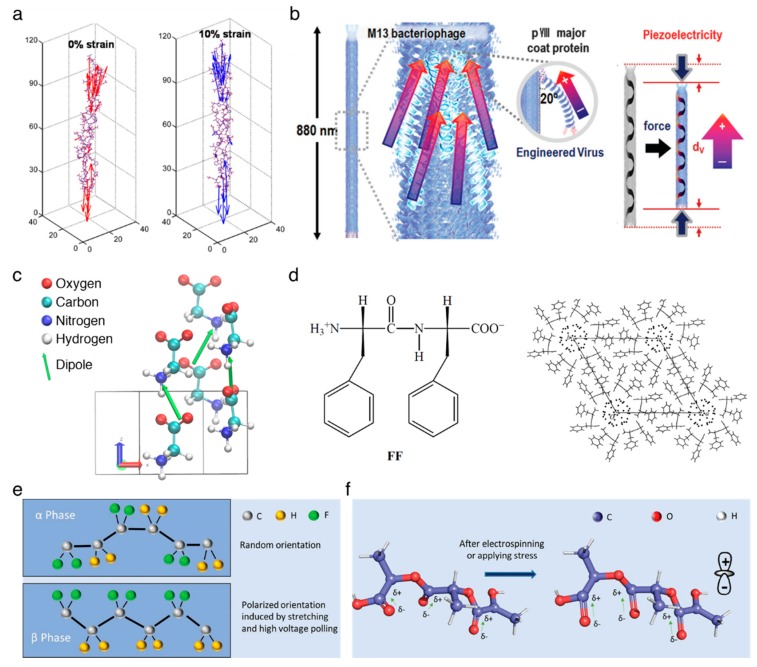
Piezoelectricity in the organic piezoelectric biomaterials. (**a**) Molecular origin of the piezoelectric effect in collagen. Reproduced with permission from [20]. Copyright American Chemical Society, 2016. (**b**) Schematic illustration of piezoelectric M13 bacteriophage. Reproduced with permission from [27]. Copyright The Royal Society of Chemistry, 2015. (**c**) Unit cell of β-glycine crystal has two molecules, where two molecular dipole moments form the net dipole moment along the *z*-axis. Reproduced with permission from [40]. Copyright Nature Publishing Group, 2019. (**d**) Unit cell and molecular packing of diphenylalanine. Reproduced with permission from [44]. Copyright Wiley–VCH, 2001. (**e**) Structures of non-piezoelectric (α-phase) and piezoelectric (β-phase) poly(vinylidene fluoride) (PVDF). Reproduced with permission from [2]. Copyright Wiley–VCH, 2018. (**f**) Molecular structure of poly(L-lactic acid) (PLLA) chain. Reproduced with permission from [2]. Copyright Wiley–VCH, 2018.

It is well known that the collagen, being a main structural protein in the extracellular matrix in the tissues, mainly causes the piezoelectric effect in bone, but its clear fundamental principle has not yet been discovered. Several hypotheses have been established to elucidate the origin of piezoelectricity in the collagen fibril [20], which involve the noncentrosymmetric structure, the existence of polar bonding at the molecular level, reorientation of the C=O–NH bond in the α-helix structure, and the polarization of hydrogen bonds in collagen [21,22,23,24]. A recent study reveals that the piezoelectric effect in collagen comes from the reorientation of, and a magnitude change, in the permanent dipoles of individual charged and polar residues towards the long axis of the collagen fibril [20], as shown in Figure 1a. The shear piezoelectric constant reported is varied from *d*_14_ = 0.2 pC/N to *d*_14_ = 2.0 pC/N for collagens impregnated by bone and tendon [25], respectively.

The M13 bacteriophage is recently emerging as the functional material for multiple applications in energy harvesting [26,27,28], chemical sensor [29,30,31], and tissue regeneration [32,33]. It is a filamentous bacterial virus with the well-defined dimension of 880 nm in length and 6.6 nm in width, consisting of single-stranded DNA which is wrapped up with 2700 copies of major proteins (pVIII) and lidded with five copies of minor proteins (pIII/pVI or pVIII/pIX) on the top or bottom ends, respectively. The major protein has an ~20° tilt angle with respect to the DNA axial direction, and is arranged with a combined five-fold rotational and two-fold screw symmetry. Each major protein holds a dipole moment directed toward the DNA axis, leading to permanent polarization in both the axial and radial direction of the phage (Figure 1b). The piezoelectric constant for radial direction is *d*_33_ = ~7.8 pm/V [26], and the constant for axial direction is improved by up to around three times as high as that of radial direction [27]. Recently, Lee et al. improved the value for axial direction up to *d*_33_ = ~26.4 pm/V by unidirectionally sticking the M13 bacteriophages [28].

### 2.2. Piezoelectric Peptides

Glycine (G) is a zwitterionic amino acid and a good model building block for investigating the process of polymorphic crystallization [34,35,36]. At ambient conditions, the crystalline glycines have three distinct structures, α-, β-, and γ-structures, where the β- and γ-structures have shear piezoelectricity due to their acentric structures [37,38,39,40]. Herein, the piezoelectricity arises from the displacement of ion in the crystal, as it does in the inorganic materials, and such displacement creates a dipole in local, and a net polarization in bulk, material, as shown in Figure 1c. The β-structured glycine has a high shear piezoelectric constant, *d*_16_ = ~190 pm/V [41], which is comparable to the normal piezoelectric constant of barium titanate (BaTiO_3_) [42].

Diphenylalanine (FF) is composed of two phenylalanine (F) amino acids and can be self-assembled into semi-crystalline peptide nanotubes and microrods, exhibiting multiple advantages including morphological diversity, functional diversity, high biocompatibility, and a high Young’s modulus [16,43]. The nanostructured diphenylalanines are widely studied piezoelectric materials that have a non-centrosymmetric hexagonal space group (P6_1_) [44]. This crystalline class serves to demonstrate their diverse physical effects including piezoelectricity, second harmonic generation, optical activity, pyroelectricity, ferroelectricity, and enantiomorphism [45] (Figure 1d). The peptide nanotubes have achieved a high shear piezoelectric constant up to *d*_15_ = ~60 pm/V [46]. In order to improve the scalability as well as the uniformity of semi-crystalline film, the unidirectionally polarized and aligned diphenylalanine nanotubes films were fabricated using the meniscus-driven self-assembly process [47], representing a similar value of *d*_15_ = ~45 pm/V to that of the highly crystalline structure. In addition, Nguyen et al. have devoted to obtaining the normal piezoelectricity (reached up to *d*_33_ = ~17.9 pm/V) of the peptide nanostructures by the vertical alignment of individual microrods [48,49].

### 2.3. Other Piezoelectric Biopolymers

The poly(vinylidene fluoride) (PVDF) films have shown one of the highest piezoelectric performances among all piezoelectric polymers found to date [50]. The PVDF has five different crystalline structures, α-, β-, γ-, δ-, and ε-structures where the β-structured PVDF has a normal piezoelectricity of *d*_33_ = −33 pC/N [51]. A dipole moment is induced perpendicular to the polymer chain in each unit of PVDF due to the presence of a branched fluorine atom with a large van der Waals radius together with the electronegativity [52,53,54]. In the β-phase, the orthorhombic crystal structure, with aligned fluorine and hydrogen branches parallel to each other, contributes to the net dipole moment and piezoelectricity [55], whereas the net dipole moment is cancelled out by the anti-parallel alignment of the dipole in the α-phase hexagonal structure [56], as depicted in Figure 1e. The nature of the negative piezoelectric effect is related to the redistribution of the electron molecular orbitals and total charges under an electrical field applied [57]. The copolymer approaches have been applied to enhance the piezoelectric constant by conjugating with trifuoroethylene (TrFE) [58,59], hexafluoropropylene (HPF) [60,61], or chlorotrifluoroethylene (CTFE) [62].

The poly(*L*-lactic acid) (PLLA) is a polymorphic polymer with excellent biodegradability and biocompatibility. The thermodynamically stable conformation is the α-crystalline structure where the dipoles introduced by carbonyl groups (C=O) are not aligned along the main polymer chain. The external stimuli, such as electrospinning (Figure 1f), allows for the dipoles to be unidirectionally oriented along the stretched direction [63], which is termed the β-crystalline structure, resulting in the shear piezoelectricity of *d*_14_ = 12 pC/N [64,65]. It is worth to note that the β-crystalline PLLA, along with decent piezoelectricity, requires no polling process due to its helical structure [3], widening the application area in the biocompatible mobile devices [66].

Natural polymers are gaining more importance owing to their biocompatibility, as recent research is aiming at the investigation of the utility of piezoelectric materials for body implantable or mountable devices. The piezoelectricity of silk arises from the combined effects of a high degree of silk II, β-sheet crystallinity, and a crystalline orientation [67]. The reported piezoelectric constant is *d*_14_ = −1.5 pC/N [67]. Cellulose, which is the most abundant natural polymer on earth, is also known to have a shear piezoelectricity of *d*_14_ = 0.2 pC/N [68].

## 3. Device Applications

### 3.1. Energy Harvesting

Self-powered electronics (SPEs) have gained an attraction as an alternative powering technology due to their independence, sustainability, and maintenance-free nature. SPEs are defined as electronics that can be operated themselves without feeding from external electrical power. In these electronics, electrical energy is provided from renewable resources, such as solar, thermal and mechanical energy. Nanogenerators that are converting mechanical to electrical energy have led the SPE field as the nanogenerators possess multiple advantages, including easy fabrication, portability, and high conversion efficiency, over conventional renewable energy technologies. Ever since the discovery of the piezoelectric and triboelectric nanogenerators (PENG and TENG, respectively) [69,70], their rapid development has shifted the paradigm for mechanical energy harvesting from fast and periodic energy resources to slow and random energy resources. A number of publications have tried over the last decade to develop the various nanogenerators possessing novel device structures and advanced materials [27,71,72,73,74,75,76]. Organic piezoelectric biomaterials have taken on an important role as the functional materials for applications in humans, being implantable and mountable nanogenerators due to their remarkable biodegradability and biocompatibility.

**Figure 2:**
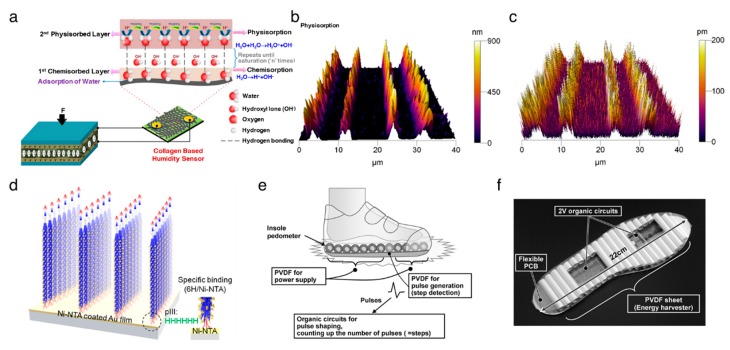
Applications of organic piezoelectric biomaterials in energy harvesting. (**a**) Schematic of sustainable energy harvesting and battery-free humidity sensor using biocompatible collagen nanofibrils. The collagen nanofibrils deposited on cotton cloth serve as a humidity sensor by measuring current signal at a fixed bias voltage. In order to demonstrate the self-powered sensing system, the energy harvester comprising collagen nanofibrils film sandwiched between Al electrodes is parallelly connected to the humidity sensor. Reproduced with permission from [77]. Copyright American Chemical Society, 2018. (**b**–**d**) Vertical self-assembly of polarized M13 bacteriophage nanostructure for energy harvesting. Reproduced with permission from [28]. Copyright American Chemical Society, 2019. (**b**) 3D-atomic force microscope (AFM) topography image of vertically aligned M13 bacteriophages. (**c**) Piezoresponse force microscope amplitude image corresponding to the 3D–AFM topography image. (**d**) The direction of polarization of the vertically aligned M13 bacteriophage with specific binding between the 6H tag on phage tail and the Ni-nitrilotriacetic acid (NTA) substrate. (**e**,**f**) Insole pedometer with piezoelectric energy harvester. Reproduced with permission from [85]. Copyright IEEE, 2012. (**e**) Schematics of the proposed insole pedometer. The pieces of PVDF sheet are used for the piezoelectric energy harvester as well as the pulse generator to detect steps in which each PVDF piece was rolled to increase the total area. The organic circuits are integrated with PVDF pieces to count the number of steps. (**f**) Photograph of the prototype insole pedometer.

Vivekananthan et al. [77] reported a piezoelectric collagen nanofibril film that is capable of both converting mechanical energy to electrical energy and functioning as a humidity sensor. A schematic of the device is illustrated in Figure 2a. Collagen-based PENG produced electrical outputs of 250 nA and 45 V. In addition, it served as a humidity sensor that showed a linear response with a good sensitivity (0.1287 μA/% RH) in the range of 50−90% room humidity. These results demonstrated a field of eco-friendly multifunctional biomaterials, towards the development of noninvasive, implantable, smart bio-medical systems.

Lee et al. [26] firstly used a self-assembled M13 bacteriophage film for a piezoelectric energy harvester in 2012. The nanogenerator yields a current of up to 6 nA and a voltage of up to 400 mV. The M13 bacteriophages are expected to have a high piezoelectric response compared to laterally assembled phages, due to their high elasticity properties along the axial direction of the DNA [78]. Shin et al. [27] reported vertically aligned M13 bacteriophage nanopillars using enforced infiltration. The vertically aligned M13 bacteriophage-based nanogenerator exhibits electrical outputs that are up to about 2.6-fold greater than those of the laterally assembled, bacteriophages-based nanogenerator. There was still a limitation on the ability to control the directionality of individual M13 bacteriophage, but Lee et al. [28] addressed this issue using genetic engineering techniques (Figure 2b–d). The resulting structure-based PENG produced up to 2.8 V of potential, 120 nA of current, and 236 nW of power from 17 N of force. The apparent versatility of the M13 bacteriophage suggests that the piezoelectric M13 bacteriophages can serve as functional nanomaterials for numerous electronic and optoelectronic applications.

Piezoelectric peptide nanostructures have also been implemented into the PENGs. Nguyen et al. [49] fabricated the vertical FF microrod arrays by applying an electric field, and then the arrays were integrated into the PENGs, representing an open-circuit voltage of 1.4 V and a power density of 3.3 nW. The performance voltage of the FF-based PENG was improved up to 2.2 V in tandem with a TENG comprising the polyethylene terephthalate and Kapton films as triboelectrically active materials [79]. Recently, Lee et al. [47] developed large-scale, unidirectionally polarized, aligned FF nanotubes and fabricated peptide-based PENGs. They used the meniscus-driven self-assembly process to fabricate horizontally aligned FF nanotubes. The fabricated FF nanotubes-based PENGs can generate voltage, current, and power of up to 2.8 V, 37.4 nA, and 8.2 nW, respectively. Hence, the FF nanostructures will act as a compatible energy source for biomedical applications in the future.

The PVDF and its copolymers have been adopted for flexible PENGs due to their inherent flexibility, high processability, and mechanical rigidity [80,81,82]. Chang et al. [83] developed a method to directly fabricate the PVDF nanofibers with a β-crystalline structure using the near-field electrospinning process, which provides a peak current of 3 nA and a peak voltage of 30 mV after integration into the PENG. A hybrid nanogenerator, demonstrated by Hansen et al. [84], is made of a piezoelectric PVDF nanofibers-based PENG and a flexible biofuel cell, and was used for powering a single nanowire-based ultraviolet sensor to build an SPEs. As shown in Figure 2e,f, Ishida et al. [85] demonstrated a self-powered pedometer, which consisted of a PVDF sheet, a 2 V organic circuit, and a flexible printed circuit board. This work suggested that the PVDF sheet can not only harvest the mechanical energy from footsteps but also serve as a footstep sensor. Sun et al. [86] and Xue et al. [87] envisioned a PVDF nanostructures-based PENG to harvest energy from human respiration. Persano et al. [88] demonstrated the textile-based PENG, featuring the highly aligned electrospun fibers of the PVDF–TrFE, exhibiting superior flexibility and mechanical robustness.

### 3.2. Sensors

**Figure 3:**
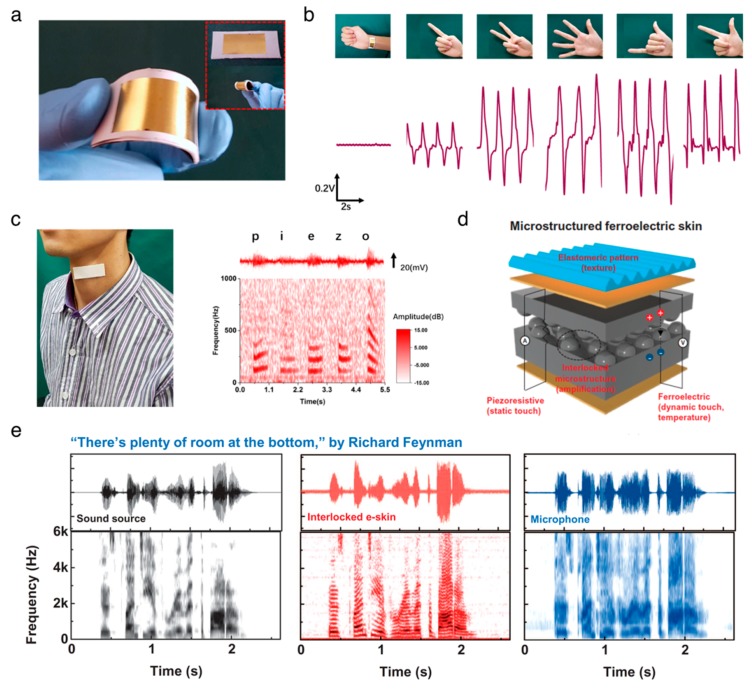
Applications of organic piezoelectric biomaterials in sensors. (**a–c**) Flexible piezoelectric nanogenerator in a wearable, self-powered active sensor for healthcare monitoring. Reproduced with permission from [89]. Copyright IOP Publishing, 2017. (**a**) Photographs of the flexible piezoelectric nanogenerator. (**b**) hand gesture sensing. (**c**) human voice recording. (**d**,**e**) Electronic skins for discriminating static/dynamic pressure stimuli. Reproduced with permission from [91]. Copyright The American Association for the Advancement of Science, 2015. (**d**) Schematic illustration of flexible and multimodal ferroelectric e-skin. (**e**) The waveform and short-time Fourier transform (STFT) signals of the sound source, readout signals from the interlocked e-skin, and microphone.

The organic biomaterials have been studied as the platform materials for biomedical pressure-sensing applications due to their high flexibility and high sensitivity to small force. The aligned PVDF–TrFE nanofibers on polyimide substrate were employed to build a flexible and lightweight pressure sensor [88]. This pressure sensor can measure small pressures down to ~0.1 Pa over the course of cyclic bending. The wearable piezoelectric PVDF sensor can serve as a healthcare monitoring device to monitor respiration signals, human gestures, and vocal cord vibrations, as demonstrated by Liu et al. [89] (Figure 3a–c). Bodkhe et al. [90] developed a pressure sensor comprised of 10% of barium titanate nanoparticle and β-crystalline phase PVDF ball mill nanocomposites using 3D printing techniques, which generated a voltage of 4 V upon gentle finger taps. The composite films of PVDF and graphene oxide developed by Park et al. [91] were used as multifunctional electronic skins to monitor multiple stimuli, including static/dynamic pressure and temperature, exhibiting a high sensitivity for monitoring simultaneous artery pulse pressures and temperature (Figure 3d,e). Recently, biodegradable and implantable sensors have gained great interest in medical applications where there is a demand for short-term functionality because biodegradable sensors are not required for the medical surgery of removal. Curry et al. [92] fabricated the piezoelectric pressure sensor featuring all the biodegradable materials of piezoelectric PLLA, molybdenum electrodes, and polylactic acid encapsulators. This device was capable of measuring a wide range of pressure, from 0 to 18 kPa. More interestingly, the sensor was completely degraded over a period of 56 days at an elevated temperature of 74 °C, indicating that this biodegradable sensor holds promise for clinical implementation. A number of publications has utilized the nature-driven piezoelectric materials to develop human physiological monitoring and electronic skins [93,94,95,96].

### 3.3. Cell and Tissue Regeneration

**Figure 4:**
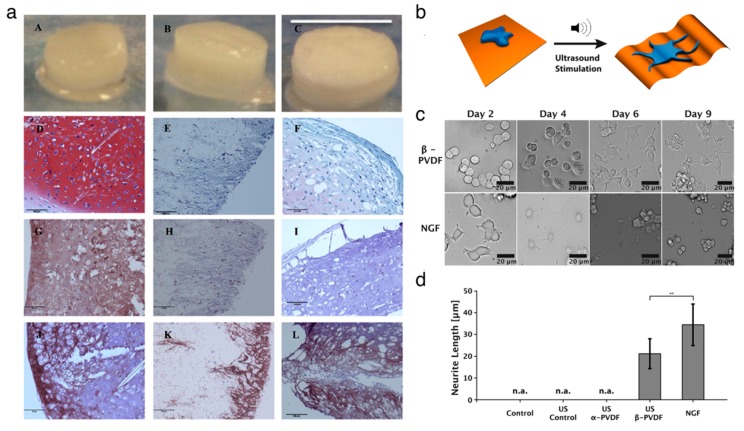
Applications of organic piezoelectric biomaterials in cell and tissue regenerations. (**a**) Representative gross images and histological images of scaffolds after 28 days undergoing chondrogenesis in dynamic conditions. As-spun PVDF–TrFE (left), annealed PVDF–TrFE (middle) and polycaprolactone (right) scaffolds. Reproduced with permission from [98]. Copyright Elsevier, 2017. (**b**–**d**) Ultrasound-mediated piezoelectric differentiation of neuron-like PC12 cells on PVDF membranes. Reproduced with permission from [100]. Copyright Nature Publishing Group, 2017. (**b**) Schematic of ultrasound stimulation of the piezoelectric β-PVDF membrane. (**c**) Comparison images of PC12 cells cultured under mechanical stimuli on PVDF substrate and neuronal growth factor stimuli. (**d**) Comparison of average neurite length of PC12 cells.

Organic piezoelectric biomaterials have been chosen as the functional materials for fabricating a scaffold to grow and differentiate cells in the field of tissue engineering [16]. Several studies have shown that the biocompatible piezoelectric materials can serve as tissue stimulators and scaffolds to promote tissue regeneration. Damaraju et al. [97] found that the cell growth on the β–phase PVDF nanofibers film exhibited higher alkaline phosphatase activity and earlier mineralization compared to the growth on random-phase PVDF film. Then, Damaraju et al. [98] showed that the 3D fibrous scaffolds decorated with electrospun PVDF–TrFE fibers stimulated the differentiation of human mesenchymal stem cells. The electromechanical actuation under high voltage helped osteogenic differentiation, whereas the actuation under low voltage aided chondrogenic differentiation (Figure 4a). Muscle cell adhesion and proliferation were improved due to the fact that the negatively charged β-phase PVDF fibers helped to elongate the muscle cells along the aligned fibers [99]. Hoop et al. [100] demonstrated that wireless stimulations helped to induce the potential in piezoelectric β-phase PVDF, improving the neurite generation in PC12 cells using the ultrasonic technique (Figure 4b–d). Similarly, the PVDF–TrFE fibril scaffolds promoted the differentiation of neural cells, neurite extension and neuronal differentiation due to the piezoelectric effect of the scaffolds [101]. The mechanical stimulation facilitates the enhanced bone cell culture on the piezoelectric PVDF substrate by applying a voltage of 5 V.

There have also been several attempts to utilize the biodegradable piezoelectric PLLA polymer as the tissue stimulator. Ikada et al. [102] intramedullary implanted PLLA rods in the cut tibiae of cats for internal fixation for up to eight weeks. The high aspect ratio of the PLLA rod enabled the enhanced fracture healing, indicated by improved callus formation, whereas the isotropic PLLA and a polyethylene control rod exhibited no effect on callus formation. Barroca et al. [103,104] observed that surface charges can change the orientation of the adsorbed proteins, resulting in the modulation of cell-binding domains. Indeed, the negatively charged PLLA improves the protein adsorption and cellular adhesion as well as proliferation.

## 4. Conclusions and Future Perspective

The present review has sought to offer insight into the importance of organic piezoelectric biomaterials in biomedical applications. We have reviewed the origin of piezoelectricity in organic piezoelectric biomaterials, including proteins, peptides, and biopolymers. The intrinsic piezoelectric property of those materials has been presented, and engineering and scientific endeavors to enhance these properties have also been reported. In summary, from current research, organic piezoelectric biomaterials have been likely to impact three major fields across many disciplines. First, they can serve as the functional materials for the power supply of implantable and mountable self-powered electronics because of their sensitivity to mechanical agitation and remarkable biocompatibility. Second, they have been utilized as platform materials for pressure sensing in biomedical applications, which is likely largely due to their high flexibility and high sensitivity to small forces in tandem with their biodegradability. Lastly, in cases where piezoelectric materials were integrated as the scaffolds for cell and tissue regeneration, those materials act as a tissue stimulator to promote the differentiation of the desired cells. An industry based on organic piezoelectric biomaterials is anticipated due to their variety of applications, but further improvements are required to smoothly implement them into practical biomedical devices.

We need to address several issues for the better integration of organic piezoelectric biomaterials into biomedical devices. Here are a few: (1) the fundamental physics of piezoelectricity in biomaterials. Even though researchers have focused on uncovering biological piezoelectricity, plenty of work remains to be done to exploit their electromechanical behavior in terms of unit cell properties. Such studies are in progress using not only single crystals of biomaterials but also calculations based on the first principle. (2) A relatively low piezoelectric constant compared to piezoelectric inorganic materials. The output performances in the applications of energy harvesting and sensors are related to the piezoelectric constant. The constant of biomaterials has been found to be much smaller than that of the state-of-the-art piezoelectric inorganic materials (*d*_33_ = 593 pC/N and d_31_ = −274 pC/N [21]), which has to be improved to achieve maximized performance. This is possible by creating proper nanostructures, aligning biomaterials, or fabricating a multilayer structure. (3) Biodegradability of the controlled manner. For biodegradable sensor and scaffold applications, the organic piezoelectric biomaterials must be decomposed within the desired time frame. The degradation rate of these materials can be engineered by different experimental treatments, such as temperature, stretching ratio, or poling electrical fields. Although researchers are still facing challenging issues, the promising physical properties of organic piezoelectric biomaterials have suggested feasible biomedical applications in energy harvesting, sensor, and tissue regeneration. We truly believe that organic piezoelectric biomaterials will continue their rapid growth in the next decade.

## Figures and Tables

**Table 1:** Comparison of piezoelectric constants for various organic piezoelectric biomaterials.

Piezoelectric Organic Biomaterials	Piezoelectric Constant		References
	Normal Piezoelectric	Shear Piezoelectric	
Collagen	-	*d*_14_ = 0.2–2.0 pC/N	[25]
M13 bacteriophage	*d*_33_ = 7.8–26.4 pm/V		[26,27,28]
Glycine	-	*d*_16_ = ~190 pm/V	[41]
Diphenylalanine	*d*_33_ = ~17.9 pm/V	*d*_15_ = 45–60 pm/V	[46,47,48,49]
PVDF	*d*_33_ = −3 pC/N *d*_31_ = 23 pC/N	-	[51]
PVDF–TrFE	*d*_33_ = −25 to −40 pC/N *d*_31_ = 12–25 pC/N	-	[52,58,59]
PVDF–HPF	*d*_33_ = −24 pC/N *d*_31_ = 30 pC/N	-	[60,61]
PVDF–CTFE	*d*_33_ = −140 pC/N	-	[62]
PLLA	-	*d*_14_ = 12 pC/N	[64,65]
Silk	-	*d*_14_ = −1.5 pC/N	[67]
Cellulose	-	*d*_14_ = 0.2 pC/N	[68]

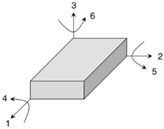
 Note: 1. The piezoelectric constant *d_ij_* is the ratio of the strain in the *j*-axis to the electric field applied along the *i*-axis. In other words, the *i* and *j* correspond to the response and excitation of materials, respectively. The inset image below depicts the coordination system. 2. The TrFE, HPF, and CTFE stand for trifuoroethylene, hexafluoropropylene, and chlorotrifluoroethylene, respectively.

## References

[1] Li C., Weng G. (2002). Antiplane crack problem in functionally graded piezoelectric materials. J. Appl. Mech..

[2] Chorsi M.T., Curry E.J., Chorsi H.T., Das R., Baroody J., Purohit P.K., Ilies H., Nguyen T.D. (2019). Piezoelectric biomaterials for sensors and actuators. Adv. Mater..

[3] Jacob J., More N., Kalia K., Kapusetti G. (2018). Piezoelectric smart biomaterials for bone and cartilage tissue engineering. Inflamm. Regen..

[4] Sirohi J., Chopra I. (1999). Fundamental understanding of piezoelectric strain sensors. J. Intell. Mater. Syst. Struct..

[5] Cannata D., Benetti M., Verona E., Varriale A., Staiano M., D’Auria S., Di Pietrantonio F. (2012). Odorant detection via Solidly Mounted Resonator biosensor. Proceedings of the 2012 IEEE International Ultrasonics Symposium.

[6] Chen D., Wang J., Xu Y. (2013). Highly sensitive lateral field excited piezoelectric film acoustic enzyme biosensor. IEEE Sens. J..

[7] Gan J., Zhang X. (2019). A review of nonlinear hysteresis modeling and control of piezoelectric actuators. AIP Adv..

[8] Dagdeviren C., Yang B.D., Su Y., Tran P.L., Joe P., Anderson E., Xia J., Doraiswamy V., Dehdashti B., Feng X. (2014). Conformal piezoelectric energy harvesting and storage from motions of the heart, lung, and diaphragm. Proc. Natl. Acad. Sci. USA.

[9] Hwang G.T., Park H., Lee J.H., Oh S., Park K.I., Byun M., Park H., Ahn G., Jeong C.K., No K. (2014). Self-powered cardiac pacemaker enabled by flexible single crystalline PMN-PT piezoelectric energy harvester. Adv. Mater..

[10] Zhu G., Wang A.C., Liu Y., Zhou Y., Wang Z.L. (2012). Functional electrical stimulation by nanogenerator with 58 V output voltage. Nano Lett..

[11] He Y.-B., Li G.-R., Wang Z.-L., Su C.-Y., Tong Y.-X. (2011). Single-crystal zno nanorod/amorphous and nanoporous metal oxide shell composites: Controllable electrochemical synthesis and enhanced supercapacitor performances. Energy Environ. Sci..

[12] Ryu J., Kim S.W., Kang K., Park C.B. (2011). Synthesis of diphenylalanine/cobalt oxide hybrid nanowires and their application to energy storage. ACS Nano.

[13] Kim D.H., Lu N., Ma R., Kim Y.S., Kim R.H., Wang S., Wu J., Won S.M., Tao H., Islam A. (2011). Epidermal electronics. Science.

[14] Choi C., Lee Y., Cho K.W., Koo J.H., Kim D.H. (2019). Wearable and implantable soft bioelectronics using two-dimensional materials. Acc. Chem. Res..

[15] Martin A.J.P. (1941). Tribo-electricity in wool and hair. Proc. Phys. Soc..

[16] Yuan H., Lei T., Qin Y., He J.H., Yang R. (2019). Design and application of piezoelectric biomaterials. J. Phys. D Appl. Phys..

[17] de Sciarra F.M. (2009). A nonlocal model with strain-based damage. Int. J. Solids Struct..

[18] Barretta R., Fabbrocino F., Luciano R., de Sciarra F.M. (2018). Closed-form solutions in stress-driven two-phase integral elasticity for bending of functionally graded nano-beams. Phys. E Low Dimens. Syst. Nanostruct..

[19] Fousek J., Cross L., Litvin D. (1999). Possible piezoelectric composites based on the flexoelectric effect. Mater. Lett..

[20] Zhou Z., Qian D., Minary-Jolandan M. (2016). Molecular mechanism of polarization and piezoelectric effect in super-twisted collagen. ACS Biomater. Sci. Eng..

[21] Bystrov V., Bdikin I., Heredia A., Pullar R., Mishina E., Sigov A., Kholkin A., Ciofani G., Menciassi A. (2012). Piezoelectricity and ferroelectricity in biomaterials: From proteins to self-assembled peptide nanotubes. Piezoelectric Nanomaterials for Biomedical Applications, Nanomedicine and Nanotoxicology.

[22] Wojnar R., Ciofani G., Menciassi A. (2012). Piezoelectric phenomena in biological tissues. Piezoelectric Nanomaterials for Biomedical Applications, Nanomedicine and Nanotoxicology.

[23] Lemanov V., Popov S., Pankova G. (2002). Piezoelectric properties of crystals of some protein aminoacids and their related compounds. Phys. Solid State.

[24] Namiki K., Hayakawa R., Wada Y. (1980). Molecular theory of piezoelectricity of α-helical polypeptide. J. Polym. Sci. Polym. Phys. Ed..

[25] Fukada E. (2000). History and recent progress in piezoelectric polymers. IEEE Trans. Ultrason. Ferroelectr. Freq. Control..

[26] Lee B.Y., Zhang J., Zueger C., Chung W.-J., Yoo S.Y., Wang E., Meyer J., Ramesh R., Lee S.-W. (2012). Virus-based piezoelectric energy generation. Nat. Nanotechnol..

[27] Shin D.-M., Han H.J., Kim W.-G., Kim E., Kim C., Hong S.W., Kim H.K., Oh J.-W., Hwang Y.-H. (2015). Bioinspired piezoelectric nanogenerators based on vertically aligned phage nanopillars. Energy Environ. Sci..

[28] Lee J.-H., Lee J.H., Xiao J., Desai M.S., Zhang X., Lee S.-W. (2019). Vertical self-assembly of polarized phage nanostructure for energy harvesting. Nano Lett..

[29] Moon J.-S., Kim W.-G., Shin D.-M., Lee S.-Y., Kim C., Lee Y., Han J., Kim K., Yoo S.Y., Oh J.-W. (2017). Bioinspired M-13 bacteriophage-based photonic nose for differential cell recognition. Chem. Sci..

[30] Moon J.-S., Lee Y., Shin D.-M., Kim C., Kim W.-G., Park M., Han J., Song H., Kim K., Oh J.-W. (2016). Identification of endocrine disrupting chemicals using a virus-based colorimetric sensor. Chem. Asian J..

[31] Kim W.-G., Zueger C., Kim C., Wong W., Devaraj V., Yoo H.-W., Hwang S., Oh J.-W., Lee S.-W. (2019). Experimental and numerical evaluation of a genetically engineered M13 bacteriophage with high sensitivity and selectivity for 2,4,6-trinitrotoluene. Org. Biomol. Chem..

[32] Shin Y.C., Kim C., Song S.-J., Jun S., Kim C.-S., Hong S.W., Hyon S.-H., Han D.-W., Oh J.-W. (2018). Ternary aligned nanofibers of RGD peptide-displaying M13 bacteriophage/PLGA/graphene oxide for facilitated myogenesis. Nanotheranostics.

[33] Raja I.S., Kim C., Song S.-J., Shin Y.C., Kang M.S., Hyon S.-H., Oh J.-W., Han D.-W. (2019). Virus-incorporated biomimetic nanocomposites for tissue regeneration. Nanomaterials.

[34] Chew J.W., Black S.N., Chow P.S., Tan R.B.H., Carpenter K.J. (2007). Stable polymorphs: Difficult to make and difficult to predict. CrystEngComm.

[35] Poornachary S.K., Chow P.S., Tan R.B.H. (2008). Influence of solution speciation of impurities on polymorphic nucleation in glycine. Cryst. Growth Des..

[36] Dowling R., Davey R.J., Curtis R.A., Han G., Poornachary S.K., Chow P.S., Tan R.B.H. (2010). Acceleration of crystal growth rates: An unexpected effect of tailor-made additives. Chem. Commun..

[37] Iitaka Y. (1954). A new form of glycine. Proc. Jpn. Soc..

[38] Kumar R.A., Vizhi R.E., Vijayan N., Babu D.R. (2011). Structural, dielectric and piezoelectric properties of nonlinear optical γ-glycine single crystals. Phys. Condens. B Matter.

[39] Iitaka Y. (1958). The crystal structure of γ-glycine. Acta Crystallogr..

[40] Guerin S., Tofail S.A.M., Thompson D. (2019). Organic piezoelectric materials: Milestones and potential. NPG Asia Mater..

[41] Guerin S., Stapleton A., Chovan D., Mouras R., Gleeson M., McKeown C., Noor M.R., Silien C., Rhen F.M.F., Kholkin A.L. (2018). Control of piezoelectricity in amino acids by supramolecular packing. Nat. Mater..

[42] Newnham R.E. (2005). Properties of Materials: Anisotropy, Symmetry, Structure.

[43] Yan X., Zhu P., Fei J., Li J. (2010). Self-assembly of peptide-inorganic hybrid spheres for adaptive encapsulation of guests. Adv. Mater..

[44] Görbitz C.H. (2001). Nanotube formation by hydrophobic dipeptides. Chem. Eur. J..

[45] Amdursky N., Beker P., Rosenman G., Aleman C., Bianco A., Venanzi M. (2013). Physics of peptide nanostructures and their nanotechnology applications. Peptide Materials: From Nanostuctures to Applications.

[46] Kholkin A., Amdursky N., Bdikin I., Gazit E., Rosenman G. (2010). Strong piezoelectricity in bioinspired peptide nanotubes. ACS Nano.

[47] Lee J.-H., Heo K., Schulz-Schönhagen K., Lee J.H., Desai M.S., Jin H.-E., Lee S.-W. (2018). Diphenylalanine peptide nanotube energy harvesters. ACS Nano.

[48] Nguyen V., Jenkins K., Yang R. (2015). Epitaxial growth of vertically aligned piezoelectric diphenylalanine peptide microrods with uniform polarization. Nano Energy.

[49] Nguyen V., Zhu R., Jenkins K., Yang R. (2016). Self-assembly of diphenylalanine peptide with controlled polarization for power generation. Nat. Commun..

[50] Nalwa H.S. (1995). Ferroelectric Polymers: Chemistry: Physics, and Applications.

[51] Someya T. (2013). Stretchable Electronics.

[52] Martins P., Lopes A.C., Lanceros-Mendez S. (2014). Electroactive phases of poly (vinylidene fluoride): Determination, processing and applications. Prog. Polym. Sci..

[53] Cui Z., Hassankiadeh N.T., Zhuang Y., Drioli E., Lee Y.M. (2015). Crystalline polymorphism in poly(vinylidenefluoride) membranes. Prog. Polym. Sci..

[54] Wan C., Bowen C.R. (2017). Multiscale-structuring of polyvinylidene fluoride for energy harvesting: The impact of molecular-, micro-and macro-structure. J. Mater. Chem. A.

[55] Lovinger A.J. (1981). Conformational defects and associated molecular motions in crystalline poly (vinylidene fluoride). J. Appl. Phys..

[56] Ameduri B. (2009). From vinylidene fluoride (VDF) to the applications of VDF-containing polymers and copolymers: Recent developments and future trends. Chem. Rev..

[57] Bystrov V.S., Paramonova E.V., Bdikin I.K., Bystrova A.V., Pullar R.C., Kholkin A.L. (2013). Molecular modeling of the piezoelectric effect in the ferroelectric polymer poly(vinylidene fluoride) (PVDF). J. Mol. Model..

[58] Lovinger A.J. (1983). Ferroelectric polymers. Science.

[59] Lutkenhaus J.L., McEnnis K., Serghei A., Russell T.P. (2010). Confinement effects on crystallization and Curie transitions of poly(vinylidene fluoride-co-trifluoroethylene). Macromolecules.

[60] Kunstler W., Wegener M., Seiss M., Gerhard-Multhaupt R. (2002). Preparation and assessment of piezo- and pyroelectric poly(vinylidene fluoride-hexafluoropropylene) copolymer films. Appl. Phys. A.

[61] Huan Y., Liu Y., Yang Y. (2007). Simultaneous stretching and static electric field poling of poly(vinylidene fluoride-hexafluoropropylene) copolymer films. Polym. Eng. Sci..

[62] Li Z., Wang Y., Cheng Z.-Y. (2006). Electromechanical properties of poly(vinylidene-fluoride-chlorotrifluoroethylene) copolymer. Appl. Phys. Lett..

[63] Sultana A., Ghosh S.K., Sencadas V., Zheng T., Higgins M.J., Middya T.R., Mandal D. (2017). Human skin interactive self-powered wearable piezoelectric bio-e-skin by electrospun poly-L-lactic acid nanofibers for non-invasive physiological signal monitoring. J. Mater. Chem. B.

[64] Tajitsu Y. (2008). Piezoelectricity of chiral polymeric fiber and its application in biomedical engineering. IEEE Trans. Ultrason. Ferroelectr. Freq. Control.

[65] Fukada E. (1998). New piezoelectric polymers. Jpn. J. Appl. Phys..

[66] Yoshida T., Imoto K., Tahara K., Naka K., Uehara Y., Kataoka S., Date M., Fukada E., Tajitsu Y. (2010). Piezoelectricity of poly(L-lactic acid) composite film with stereocomplex of poly(L-lactide) and poly(D-lactide). Jpn. J. Appl. Phys..

[67] Yucel T., Cebe P., Kaplan D.L. (2011). Structural origins of silk piezoelectricity. Adv. Funct. Mater..

[68] Kim J., Yun S., Ounaies Z. (2006). Discovery of cellulose as a smart material. Macromolecules.

[69] Wang Z.L., Song J. (2006). Piezoelectric nanogenerators based on zinc oxide nanowire arrays. Science.

[70] Fan F.-R., Tian Z.-Q., Wang Z.L. (2012). Flexible triboelectric generator. Nano Energy.

[71] Kim G.H., Shin D.-M., Kim H.-K., Hwang Y.-H. (2015). Effect of the dielectric layer on the electrical output of a ZnO nanosheet-based nanogenerator. J. Korean Phys. Soc..

[72] Kim T., Jeon S., Lone S., Doh S.J., Shin D.-M., Kim H.K., Hwang Y.-H., Hong S.W. (2018). Versatile nanodot-patterned Gore-Tex fabric for multiple energy harvesting in wearable and aerodynamic nanogenerators. Nano Energy.

[73] Park H.-Y., Kim H.K., Hwang Y.-H., Shin D.-M. (2018). Water-through triboelectric nanogenerator based on Ti-mesh for harvesting liquid flow. J. Korean Phys. Soc..

[74] Phan H., Shin D.-M., Jeon S.H., Kang T.Y., Han P., Kim G.H., Kim H.K., Kim K., Hwang Y.-H., Hong S.W. (2017). Aerodynamic and aeroelastic flutters driven triboelectric nanogenerators for harvesting broadband airflow energy. Nano Energy.

[75] Shin D.-M., Tsege E.L., Kang S.H., Seung W., Kim S.-W., Kim H.-K., Hong S.W., Hwang Y.-H. (2015). Freestanding ZnO nanorod/graphene/ZnO nanorod epitaxial double heterostructure for improved piezoelectric nanogenerators. Nano Energy.

[76] Tsege E.L., Shin D.-M., Lee S., Kim H.-K., Hwang Y.-H. (2016). Highly Durable Ti-mesh based triboelectric nanogenerator for self-powered device applications. J. Nanosci. Nanotechnol..

[77] Vivekananthan V., Alluri N.R., Purusothaman Y., Chandrasekhar A., Selvarajan S., Kim S.-J. (2018). Biocompatible collagen nanofibrils: An approach for sustainable energy harvesting and battery-free humidity sensor applications. ACS Appl. Mater. Interfaces.

[78] Shi Y., He S., Hearst J.E. (1996). Statistical mechanics of the extensible and shearable elastic rod and of DNA. J. Chem. Phys..

[79] Vu N., Kelly S., Yang R. (2017). Piezoelectric peptide-based nanogenerator enhanced by single-electrode triboelectric nanogenerator. APL Mater..

[80] Fang J., Wang X., Lin T. (2011). Electrical power generator from randomly oriented electrospun poly(vinylidene fluoride) nanofibre membranes. J. Mater. Chem..

[81] Pan C.-T., Yen C.-K., Wang S.-Y., Lai Y.-C., Lin L., Huang J.C., Kuo S.-W. (2015). Near-field electrospinning enhances the energy harvesting of hollow PVDF piezoelectric fibers. RSC Adv..

[82] Liu Z.H., Pan C.T., Lin L.W., Huang J.C., Ou Z.Y. (2014). Direct-write PVDF nonwoven fiber fabric energy harvesters via the hollow cylindrical near-field electrospinning process. Smart Mater. Struct..

[83] Chang C., Tran V.H., Wang J., Fuh Y.-K., Lin L. (2010). Direct-write piezoelectric polymeric nanogenerator with high energy conversion efficiency. Nano Lett..

[84] Hansen B.J., Liu Y., Yang R., Wang Z.L. (2010). Hybrid nanogenerator for concurrently harvesting biomechanical and biochemical energy. ACS Nano.

[85] Ishida K., Huang T.-C., Honda K., Shinozuka Y., Fuketa H., Yokota T., Zschieschang U., Klauk H., Tortissier G., Sekitani T. (2013). Insole pedometer with piezoelectric energy harvester and 2 V organic circuits. IEEE J. Solid State Circuits.

[86] Sun C., Shi J., Bayerl D.J., Wang X. (2011). PVDF microbelts for harvesting energy from respiration. Energy Environ. Sci..

[87] Xue H., Yang Q., Wang D., Luo W., Wang W., Lin M., Liang D., Luo Q. (2017). A wearable pyroelectric nanogenerator and self-powered breathing sensor. Nano Energy.

[88] Persano L., Dagdeviren C., Su Y., Zhang Y., Girardo S., Pisignano D., Huang Y., Rogers J.A. (2013). High performance piezoelectric devices based on aligned arrays of nanofibers of poly (vinylidenefluoride-co-trifluoroethylene). Nat. Commun..

[89] Liu Z., Zhang S., Jin Y.M., Ouyang H., Zou Y., Wang X.X., Xie L.X., Li Z. (2017). Flexible piezoelectric nanogenerator in wearable self-powered active sensor for respiration and healthcare monitoring. Semicond. Sci. Technol..

[90] Bodkhe S., Turcot G., Gosselin F.P., Therriault D. (2017). One-step solvent evaporation-assisted 3D printing of piezoelectric PVDF nanocomposite structures. ACS Appl. Mater. Interfaces.

[91] Park J., Kim M., Lee Y., Lee H.S., Ko H. (2015). Fingertip skin-inspired microstructured ferroelectric skins discriminate static/dynamic pressure and temperature stimuli. Sci. Adv..

[92] Curry E.J., Ke K., Chorsi M.T., Wrobel K.S., Miller A.N., Patel A., Kim I., Feng J., Yue L., Wu Q. (2018). Biodegradable piezoelectric force sensor. Proc. Natl. Acad. Sci. USA.

[93] Joseph J., Singh S.G., Vanjari S.R.K. (2017). Leveraging innate piezoelectricity of ultra-smooth silk thin films for flexible and wearable sensor applications. IEEE Sens. J..

[94] Wang X., Gu Y., Xiong Z., Cui Z., Zhang T. (2014). Silk-molded flexible, ultrasensitive, and highly stable electronic skin for monitoring human physiological signals. Adv. Mater..

[95] Ghosh S.K., Mandal D. (2017). Sustainable energy generation from piezoelectric biomaterial for noninvasive physiological signal monitoring. ACS Sustain. Chem. Eng..

[96] Moreno S., Baniasadi M., Mohammed S., Mejia I., Chen Y., Quevedo-Lopez M.A., Kumar N., Dimitrijevich S., Minary-Jolandan M. (2015). Flexible electronics: Biocompatible collagen films as substrates for flexible implantable electronics. Adv. Electron. Mater..

[97] Damaraju S.M., Wu S., Jaffe M., Arinzeh T.L. (2013). Structural changes in pvdf fibers due to electrospinning and its effect on biological function. Biomed. Mater..

[98] Damaraju S.M., Shen Y., Elele E., Khusid B., Eshghinejad A., Li J., Jaffe M., Arinzeh T.L. (2017). Three-dimensional piezoelectric fibrous scaffolds selectively promote mesenchymal stem cell differentiation. Biomaterials.

[99] Martins P.M., Ribeiro S., Ribeiro C., Sencadas V., Gomes A.C., Gama F.M., Lancerosmendez S. (2013). Effect of poling state and morphology of piezoelectric poly(vinylidene fluoride) membranes for skeletal muscle tissue engineering. RCS Adv..

[100] Hoop M., Chen X.Z., Ferrari A., Mushtaq F., Ghazaryan G., Tervoort T., Poulikakos D., Nelson B., Pane S. (2017). Ultrasound-mediated piezoelectric differentiation of neuron-like PC12 cells on PVDF membranes. Sci. Rep..

[101] Lee Y.S., Arinzeh T.L. (2012). The influence of piezoelectric scaffolds on neural differentiation of human neural stem/progenitor cells. Tissue Eng. Part A.

[102] Ikada Y., Shikinami Y., Hara Y., Tagawa M., Fukada E. (1996). Enhancement of bone formation by drawn poly(L-lactide). J. Biomed. Mater. Res. Part A.

[103] Barroca N., Daniel-da-Silva A., Gomes P., Fernandes M., Lanceros-Mendez S., Sharma P., Gruverman A., Fernandes M., Vilarinho P. (2012). Suitability of PLLA as piezoelectric substrates for tissue engineering evidenced by microscopy techniques. Microsc. Microanal..

[104] Barroca N., Vilarinho P.M., Daniel-da-Silva A.L., Wu A., Fernandes M.H., Gruverman A. (2011). Stability of electrically induced-polarization in poly (L-lactic) acid for bone regeneration. Appl. Phys. Lett..

